# Endoscopic Management of Biliary Strictures after Orthotopic Liver Transplantation: A Single Center Experience Study

**DOI:** 10.3390/diagnostics12051221

**Published:** 2022-05-13

**Authors:** Vasile Sandru, Madalina Stan-Ilie, Oana-Mihaela Plotogea, Catalina Vladut, Bogdan Silviu Ungureanu, Gheorghe G. Balan, Dan Ionut Gheonea, Gabriel Constantinescu

**Affiliations:** 1 Department of Gastroenterology, Clinical Emergency Hospital of Bucharest, 105402 Bucharest, Romania; drsandruvasile@gmail.com (V.S.); drmadalina@gmail.com (M.S.-I.); plotogea.oana@gmail.com (O.-M.P.); gabrielconstantinescu63@gmail.com (G.C.); 2 Department of Gastroenterology, University of Medicine and Pharmacy of Craiova, 200349 Craiova, Romania; digheonea@gmail.com; 3 Department 5, “Carol Davila” University of Medicine and Pharmacy, 050474 Bucharest, Romania; 4 Department of Gastroenterology, Prof. Dr. Agrippa Ionescu Clinical Emergency Hospital, 011356 Bucharest, Romania; drcatalinavladut@gmail.com; 5 Department of Gastroenterology, “Grigore T. Popa” University of Medicine and Pharmacy Iasi, 700115 Iasi, Romania; balan.gheo@yahoo.com; 6 Institute of Gastroenterology and Hepatology, “St. Spiridon” Emergency Hospital, 700111 Iasi, Romania

**Keywords:** orthotopic liver transplantation (OLT), biliary strictures, fully covered self-expandable metal stents (FCSEMS), multiple plastic stents (MPS), therapeutic endoscopy

## Abstract

Background and Aim. Endoscopic therapy is the method of choice in the management of biliary strictures after orthotopic liver transplantation (OLT). Even though the mainstay approach for OLT stricture complications is represented by consecutive procedures of multiple plastic stents (MPS) insertion, a valuable alternative is the use of fully covered self-expandable metal stents (FCSEMS). The aim of the study was to compare MPS with FCSEMS used in the management of OLT biliary strictures, in terms of clinical outcomes and complications. Material and Methods. This is a retrospective, single-center study conducted between February 2014 and November 2019 in the Clinical Emergency Hospital of Bucharest, Romania. We enrolled all consecutive patients who developed biliary strictures after OLT and were treated by endoscopic retrograde cholangiopancreatography (ERCP) either with MPS or FCSEMS. Results. Thirty-six patients were included in the study, 27 patients had MPS and nine patients had FCSEMS. 106 ERCP procedures were performed and 159 stents were inserted. The mean number of ERCP procedures required per patient was significantly higher for MPS than for FCSEMS (3.34 ± 1.46 vs. 2.11 ± 0.33, *p* < 0.001). In the FCSEMS group only one patient had three procedures, due to stent migration. Difficult cannulation was encountered in 22 patients, 18 patients from MPS group and four patients from FCSEMS group. Dilation was performed in 20 (77%) MPS patients and in four FCSEMS patients (44%). Overall, we used 159 stents for stricture resolution, 149 plastic stents and 10 FCSEMS. Mean number of stents inserted was significantly lower in the FCSEMS group compared to MPS group (1.16 ± 0.40 vs. 5.73 ± 2.64, *p* < 0.001). Conclusions. Stricture resolution using FCSEMS is comparable to MPS and even has some advantages. In line with prior studies, FCSEMS are effective, with fewer complications and similar outcome compared to plastic stents. Other particular aspects should be further assessed, especially long-term follow up of FCSEMS and their cost efficiency.

## 1. Introduction

Orthotopic liver transplantation (OLT) is considered the curative option for end-stage liver disease [1]. Regardless of all advances in surgical techniques for OLT, recipient, and donor criteria, biliary complications are still common. Biliary anastomotic strictures occur in almost 40% of patients that undergo OLT, especially in patients that receive the organ from a living donor [2].

Endoscopic therapy is the preferred therapeutic management for biliary strictures, with significant improvements over the last decade. Currently, the optimal strategy for benign biliary strictures consists of balloon dilation along with sequential placement of multiple plastic stents (MPS) [3]. A single ERCP session is not considered effective for stricture resolution, mostly because of the fibrotic tissue which develops after end-to-end choledochocholedochostomy, thus, requiring additional interventions. Multiple admissions are necessary and create physical and emotional discomfort for the patient, as well as high costs after every procedure [4,5,6].

Fully covered self-expandable metal stents (FCSMES) are considered a viable option for malignant strictures. Moreover, these stents have recently shown promising results for benign biliary strictures. Placing a single expandable stent might be more appealing than using at least three side-to-side plastic stents, which also require replacement every three months. However, FCSEMS seem to have a high rate of migration which may limit their benefits [7,8].

Several studies have shown the safety of FCSEMS for OLT patients with similar results after MPS stents; however, there is limited comparative evidence, especially due to the limited number of patients [9,10,11]. Hereby, we present our experience in terms of efficacy and safety, regarding endoscopic therapy in patients with post-OLT biliary strictures. Thus, the aim our study was to assess the clinical outcome, including complications, by comparing plastic stents with metal stents (FCSEMS).

## 2. Materials and Methods

This is a retrospective, single-center study conducted between February 2014 and November 2019. We included in the study all consecutive patients who developed biliary strictures after surgical interventions for OLT. All endoscopic procedures were performed within the Clinical Emergency Hospital of Bucharest, Romania, which represents a high-volume center for ERCP procedures (endoscopic retrograde cholangiopancreatography) with an average of 1700 procedures/year.

### 2.1. Patients

All patients were referred by the transplant team or presented in the emergency room and admitted to our department. Diagnosis of biliary stricture was well documented based on the biologic status and after biliary imaging. Informed consent was obtained from every patient before ERCP procedures and all interventions were performed in accordance with the World Medical Association Declaration of Helsinki Ethical Principals.

We collected data regarding age, gender, transplantation indication, year of transplant, and first stenotic episode. Other technical parameters were also taken into consideration: types of anastomoses performed, difficulty of biliary cannulation, dilation, number of required ERCP procedures, number of stents used per patient, types of stents and their characteristics. Patients were followed starting with stents’ placement until their removal.

We only included patients at their first episode, with no ERCP procedure performed before.

### 2.2. Procedure

After stricture confirmation by biliary imaging, all included patients underwent an ERCP procedure by an experienced endoscopist with a TJF-Q180V duodenoscope (Olympus, Tokyo, Japan) under propofol sedation.

After biliary cannulation, we performed cholangiogram to confirm the anastomotic stricture. This was defined as an isolated narrowing of the bile duct with benign characteristics, occluding more than 70% of the biliary lumen associated or not with dilation of intrahepatic ducts. The next step consisted in passing a guidewire (JagwireTM Boston Scientific), followed by balloon dilation if considered necessary.

FCSEMS and MPS were used according to the endoscopist’s decision. When FCSEMS were considered, we first measured the distance between the papilla and anastomotic stricture to choose the correct length. Its position should be 1 cm above the stricture and no more than 1 cm within the duodenum lumen. All FCSEMS were provided by Boston Scientific Marlborough, Massachusetts, USA (WallFlex Fully Covered Stents) and had a 10 mm diameter with a length of 60 to 80 mm. For MPS, we chose multiple stent insertion (Boston Scientific Flexima, mainly preloaded stents), reaching the maximum number of stents that could have been inserted, mostly using a 10 Fr caliber. Patients were followed with ERCP re-check scheduled for each patient. If stent migration or occlusion was suspected, ERCP was immediately performed.

### 2.3. Outcomes

The anastomotic stricture was assessed after FCSEMS or MPS placement regarding stricture resolution. Stricture resolution was defined after follow-up by performing a cholangiogram and passing a 12 mm extraction balloon through the former stricture. Any complications during or immediately after ERCP were also noted.

### 2.4. Statistical Analysis

Patients were divided into two groups, according to the type of stent used: group 1—plastic stents and group 2—metal stents. For statistical analysis, we used IBM SPSS 20.0 v.20 software package and considered statistically significant a *p* value below 0.05.

## 3. Results

### 3.1. Patients’ Characteristics

A total of 39 patients were assessed for eligibility. Thirty-six patients were ultimately included in the study. Three patients refused the procedure. 27 patients had MPS and nine patients had FCSEMS.

ERCP was successfully performed in 35/36 (97.2%). One patient died of massive haemobilia, 2 days after plastic stent placement.

The mean age of patients with MPS was 53.17 years while, in the group of patients with FCSEMS, this was 60.67 years. In relation to gender, 33.3% of the patients with MPS were female and 66.7% were male, while 16.7% of the patients with expandable stents were female and 83.3% were male. Comparing the group of patients with MPS and the group of patients with an expandable stent, a *p* value > 0.05 was found (Table 1), indicating that there was no statistically significant difference in relation to age or gender. Therefore, the gender distribution was similar in both groups with no differences regarding their age.

All patients included in the study received a deceased donor liver and the main cause for liver transplant was viral disease, either HBV, HCV, or related (57%), followed by alcoholic liver cirrhosis (25.7%). (Table 2). All patients underwent choledochocholedochostomy and all strictures were anastomotic strictures.

### 3.2. Procedures’ Outcomes

Between February 2014 and November 2019, 106 ERCPs were performed and 159 stents were inserted for the treatment of post-transplant biliary stenoses. All patients in the FCSEMS group, underwent biliary sphincterotomy, whereas this procedure was carried out for only 18 patients that had MPS.

Overall mean number of ERCP procedures required per patient was 3.34 ± 1.46 for MPS and 2.11 ± 0.33 for FCSEMS (*p* < 0.001). In the FCSEMS group, only one patient had three procedures, due to stent migration. Difficult cannulation was encountered in 18 patients with 70% of the MPS group and 44% of the FCSEMS group. Dilation was performed in both groups of patients when considered necessary. For the MPS group, dilation was used in 20 patients (77%) and in the FCSEMS group in four patients (44%). Overall, we used 159 stents for strictures’ resolution, 149 plastic stents and 10 FCSEMS, respectively. The mean number of stents used in the FCSEMS group was significantly lower than that used in the MPS group (1.16 ± 0.40 vs. 5.73 ± 2.64, *p* < 0.001) (Table 3).

**Figure 1:**
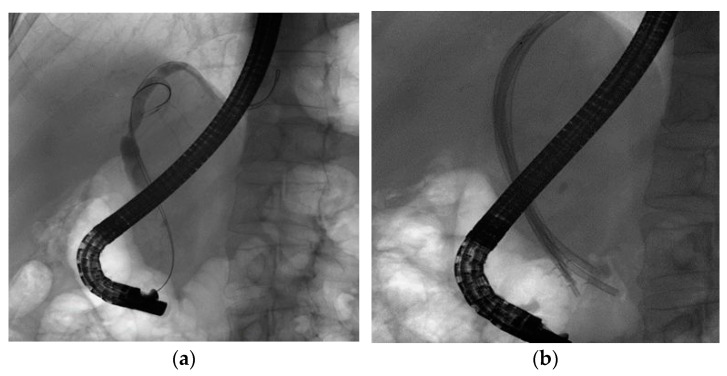
Cholangiogram of hilar biliary stenosis post OLT before (**a**) and after (**b**) multiple plastic stent insertion.

**Figure 2:**
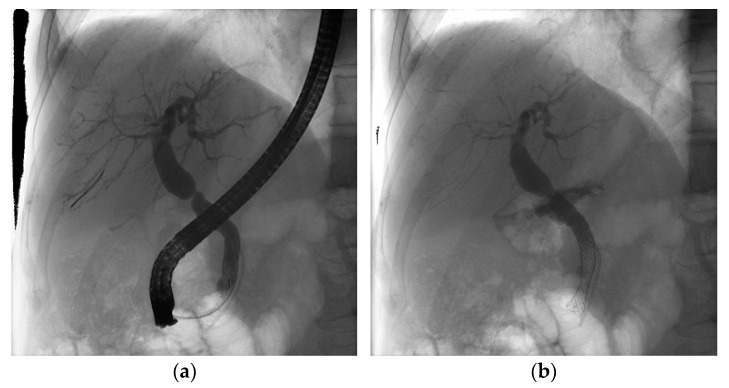
Cholangiogram of hilar biliary stenosis post OLT before (**a**) and after (**b**) expandable stent insertion.

Stricture resolution (Figure 1 and Figure 2) has been documented in all nine cases with FCSEMS placement, with a mean time of stent removal of 7.43 months. SEMS were never extracted before 6 months. On the other hand, for MPS we could confirm stent resolution only for 18 cases because the other eight patients did not refer further to our clinic for follow-up. The mean MPS time for stricture resolution of stent removal was 6.32 months. Plastic stents were electively changed every 3 months or earlier whenever necessary. Taking into consideration that the subjects are immunosuppressed patients, they are closely followed up by the transplant team (the surgeon that performed the transplant and a gastroenterologist from the transplant center). The transplant team further sends the patient for stent exchange when there are very early of signs of obstruction and/or clinical and biochemical abnormalities.

### 3.3. Complications

Stent placement was successful in all 36 patients. Adverse events occurred in 37.03% of patients that underwent MPS and 22.2% following FCSEMS. However, three patients treated with MPS noted pain, four patients experienced secondary ERCP pancreatitis and one patient had hemobilia after the first ERCP procedure. We also had two patients with migrated plastic stents after the first ERCP session. One patient developed mild pancreatitis after FCSEMS placement. In the FCSEMS group we had one case of stent migration, which was solved by placing another FCSEMS. We did not encounter any cases with occlusion caused by stent migration.

## 4. Discussion

The endoscopic approach has been embedded as the standard of care for biliary anastomotic complications after liver transplant (LT). The transition from surgery or percutaneous transhepatic procedures to ERCP for biliary strictures has succeeded in a lower rate of complications. Plastic stents along with balloon dilation are widely used for biliary stricture resolution with a high success rate [12]. Liver transplantation covers both deceased and living donor recipients. When compared side by side, living donor LT has a considerably higher risk of stricture than deceased donor LT since the duct-to-duct reconstruction involves smaller bile ducts [13,14]. This requires a more complex procedure with the anastomosis between the small intrahepatic duct of the liver donor and the common bile duct of the recipient. Thus, the anastomosis is related to a narrow-angle, and bile duct stricture occurs more frequently. Balloon dilation and placement of MPS are used to surpass this complication by remodeling the bile duct and allowing biliary drainage [15]. Traditionally considered landmarks for malignant strictures, FCSEMS have been adopted in post-LT biliary strictures resolution as new alternatives. FCSEMS seem to have a technical advantage over MPS, since the anastomotic graft provides enough space above the stricture, thus the stent might be well deployed and positioned further from the hepatic confluence [16,17,18]. Nevertheless, there are several studies in the literature that could not suggest an obvious advantage of self-expandable metal stents over multiple insertions of plastic stents in terms of stricture resolution rates, in addition indicating similar overall rates of complications, including stent migration [7,19,20,21].

Most of the ERCP procedures we performed were noted within the MPS patients. The use of MPS generally requires multiple procedures to achieve full stricture resolution. A study revealed a stricture resolution of 44% which seemed to be related not only to the anastomotic complication but also to the lower number of PS used, as just 1 PS was used in the ERCP. Our results are closely similar to the available data from all meta-analyses published so far, with a resolution rate of 94,5% for PS and 100% resolution rate for FCSEMS [22]. However, we did not proceed in a longer follow-up of patients, and we considered stricture resolution by performing a cholangiography and balloon extraction passing just after stent removal. Thus, we could not provide any data, regarding the recurrence rate. In an observational study conducted by Poley et al. during 5-year follow-up, the chance of remaining stent-free after FCSEMS removal was 48.9% among the entire cohort, concluding that FCSEMS represent a viable long-term option for biliary strictures post-LT [20]. Moreover, the probability of not having stricture recurrence among subjects with stricture resolution at the moment of FCSEMS removal was 73% [23].

There are several factors that have been discussed in the scientific literature regarding the poor outcome and the stricture recurrence. For example, tight strictures might represent a technical challenge for cannulation with a guidewire, while delayed presentation, after more than 6 months is associated with a higher rate of recurrence. Other risk factors for stricture recurrence are bile leakages at initial ERCP and presence of T tubes, which have been widely used in the past [24,25].

As concerns the number of stents and ERCP procedures, we expected this to be higher in the MPS patients, since they require at least three stents and a shorter time for stent changing. This is a major drawback when compared to FCSEMS, which basically requires only placing and removing of the stent. Our results showed a high discrepancy between the two groups’ data with high statistical significance, with only one patient receiving three ERCP procedures in the SEMS group secondary to stent migration.

Regarding treatment length, some studies have suggested maintaining the MPS for up to 12 months to assure a lower chance of recurrence [26,27]. The mean time for the final ERCP for MPS in our study was 7.43 months; however, we believe this was related to patient compliance for repeated ERCP, since every procedure was carefully scheduled. Martins et al. [8] suggested that a follow-up period of nearly 3 years is necessary to assess stricture recurrence. As for the FCSEMS we had a mean period for the last ERCP of 6.32 months. Even though others have suggested an even more prolonged time for FCSEMS, we successfully obtained stricture resolution at the last ERCP.

Stent migration remains a common issue. FCSEMS distal migration represents the major challenge that is frequently discussed. While Kaffes et al. [27] used a specific stent with a wider diameter at the ends to avoid migration, others have reported spontaneous migration. In order to prevent stent migration, several endoscopists placed the SEMS entirely inside the common bile duct [26]. We reported only on FCSEMS migration in a patient where the stent was not fully placed within the CBD and was replaced, since the stricture was not resolved at that time. Post-procedure pancreatitis did not occur in the FCSEMS patients, perhaps because all included patients had sphincterotomy, regardless of the risk of migration. On the other hand, we encountered two patients who developed mild pancreatitis after MPS placement, and some also presented with pain, thus requiring analgesics. Other complications noted by various researchers were cholangitis and hematemesis early after stent deployment [28].

FCSEMS are associated with a lower number of procedures, thus, diminishing the infection rate and proving to be more cost-efficient [21,29]. A limitation of the study was that we did not perform a cost analysis, since it was a retrospective study, and there was no time-lapse monitoring for all patients. However, based on our experience, there is a financial benefit when using FCSEMS, since we performed a lower number of procedures, which emphasizes that additional admission for more ERCPs procedures is at stake when using MPS.

Other limitations inherent in this study should be noted. This was retrospective, non-randomized, and with a small number of patients, especially in the FCSEMS group. We could not assess patients’ follow-up as we had suboptimal treatment management for MPS compared to other available studies and we could not provide a long period of time to assess the potential recurrence rate. Moreover, future studies should consider a thorough analysis of cost-efficiency and new prothesis development to avoid possible complications and to assess stent indwelling time [30].

The strength of this study consists in the fact that it is the first paper in Romania that presents original and unique data regarding endoscopic treatment in patients with anastomotic benign strictures following liver transplantation.

## 5. Conclusions

Stricture resolution using FCSEMS is comparable to MPS and even has some advantages. Our research showed that FCSEMS are effective, requiring fewer procedures, thus providing more comfort for the patient. Therefore, we recommend that whenever it is technically feasible, FCSEMS should be the first choice for post OLT stenosis. Other particular aspects should be further assessed, especially long-term follow-up of FCSEMS and their cost-efficiency.

## Figures and Tables

**Table 1:** Patients’ characteristics.

	Plastic Stents	Metal Stents	
Variables	Mean (±Standard Deviation)/ Number (Percentage)	Mean (±Standard Deviation)/ Number (Percentage)	*p*-Value
Age	53.17 (±12.36)	60.67 (±10.46)	0.074
Gender	F (n = 10, 33.3%)	F (n = 1, 16.7%)	0.643
	B (n = 20, 66.7%)	B (n = 5, 83.3%)	

**Table 2:** The patients’ indication for liver transplant and the stents used.

Indication for Liver Transplant		Number of Patients According to Type of Stent, n (%)	
	Plastic Stents	Metal Stents	Total
Alcoholic liver cirrhosis	7 (25.9%)	3 (33%)	10 (27.7%)
Liver cirrhosis HBV + HDV	4 (15%)	0	4 (11.4%)
Liver cirrhosis HCV	4 (15%)	2 (22%)	6 (17%)
Cryptogenic hepatic cirrhosis	2 (7.7%)	1 (11%)	3 (8.6%)
Liver cirrhosis HBV	3 (11.5%)	0	3 (8.6%)
HCC graft on liver cirrhosis HBV + HDV	2 (7.7%)	2 (22%)	4 (11.5%)
Budd Chiari Syndrome	1 (3.9%)	0	1 (2.8%)
Autoimmune liver cirrhosis	0	1 (11%)	1 (2.8%)
Caroli Syndrome	1 (3.9%)	0	1 (2.8%)
Liver cirrhosis HBV + HCV	1 (3.9%)	0	1 (2.8%)
Liver cirrhosis HBV+ Alcoholic liver cirrhosis	1 (3.9%)	0	1 (2.8%)
HCC graft on liver cirrhosis HBV	1 (3.9%)	0	1 (2.8%)
Total	27	9	36

Legend. HBV = hepatitis B virus; HDV = hepatitis D virus; HCV = hepatitis C virus; HCC = hepatocarcinoma.

**Table 3:** Comparisons between MPS and FCSEMS procedures’ characteristics.

	MPS	FCSEMS	*p*-Value
Total number of ERCP procedures	87	19	
Number of ERCP per patient			
Mean (±SD)	3.34 (±1.46)	2.11 (±0.33)	<0.001
Median	3	2	
Range	2–8	2–3	
Total number of stents	149	10	
Number of stents per patient			
Mean (±SD)	5.73 (±2.64)	1.16 (±0.40)	<0.001
Median	6	1	
Range	3–13	1–2	
Dilation			0.317
Yes	20 (77%)	4 (44%)	
No	6 (23%)	5 (45%)	
Difficult cannulation			0.391
Yes	18 (70%)	4 (44%)	
No	8 (30%)	5 (45%)	

## Data Availability

The data presented in this study are available on request from the first author (V.S.).
